# CcpA Regulates *Staphylococcus aureus* Biofilm Formation through Direct Repression of Staphylokinase Expression

**DOI:** 10.3390/antibiotics11101426

**Published:** 2022-10-17

**Authors:** Mingxia Zheng, Keting Zhu, Huagang Peng, Weilong Shang, Yan Zhao, Shuguang Lu, Xiancai Rao, Ming Li, Renjie Zhou, Gang Li

**Affiliations:** 1 Department of Emergency Medicine, Xinqiao Hospital, Army Medical University, Chongqing 400037, China; 2 Department of Microbiology, College of Basic Medical Sciences, Army Medical University, Chongqing 400038, China

**Keywords:** *Staphylococcus aureus*, biofilm, catabolite control protein A (CcpA), staphylokinase (Sak)

## Abstract

*Staphylococcus aureus* represents a notorious opportunistic pathogen causing various infections in biofilm nature, imposing remarkable therapeutic challenges worldwide. The catabolite control protein A (CcpA), a major regulator of carbon catabolite repression (CCR), has been recognized to modulate *S. aureus* biofilm formation, while the underlying mechanism remains to be fully elucidated. In this study, the reduced biofilm was firstly determined in the *ccpA* deletion mutant of *S. aureus* clinical isolate XN108 using both crystal violet staining and confocal laser scanning microscopy. RNA-seq analysis suggested that *sak*-encoding staphylokinase (Sak) was significantly upregulated in the mutant ∆*ccpA*, which was further confirmed by RT-qPCR. Consistently, the induced Sak production correlated the elevated promoter activity of *sak* and increased secretion in the supernatants, as demonstrated by P*sak*-*lacZ* reporter fusion expression and chromogenic detection, respectively. Notably, electrophoretic mobility shift assays showed that purified recombinant protein CcpA binds directly to the promoter region of *sak*, suggesting the direct negative control of *sak* expression by CcpA. Double isogenic deletion of *ccpA* and *sak* restored biofilm formation for mutant ∆*ccpA*, which could be diminished by *trans*-complemented *sak*. Furthermore, the exogenous addition of recombinant Sak inhibited biofilm formation for XN108 in a dose-dependent manner. Together, this study delineates a novel model of CcpA-controlled *S. aureus* biofilm through direct inhibition of *sak* expression, highlighting the multifaceted roles and multiple networks regulated by CcpA.

## 1. Introduction

Bacterial biofilm, a multicellular lifestyle with bacteria embedded in an extracellular polymeric substance, was firstly termed in 1978 [1], and predominates in various natural and clinical settings [2]. In general, the majority of bacteria has the inherent repertoire to produce and grow in this self-generated and highly structured matrix, and biofilm formation is estimated to be involved in 65%~80% of bacterial infections in humans [3,4], making it great concern for public health. *Staphylococcus aureus* constitutes a common inhabitant of the human microbiota, but also represents a notorious, opportunistic pathogen responsible for community-acquired and hospital-acquired infections worldwide. *S. aureus* can cause different types of infections, ranging from mild skin and soft tissue infections to more serious and life-threatening infections, such as bacteremia, infective endocarditis, pneumonia, and osteomyelitis [5,6]. Furthermore, *S. aureus* forms robust biofilms on both abiotic and biotic surfaces, and the switch between single free-floating cells and multicellular biofilms is critical for *S. aureus* to establish infections in vivo, particularly for biofilm-associated infections on indwelling medical devices [3,7]. The structured biofilm could protect the embedded *S. aureus* cells against hostile conditions, i.e., evasion of host immune system, and enhanced resilience to antimicrobials, imposing remarkable therapeutic challenges in clinics globally [8].

Typically, the *S. aureus* biofilm is characterized by three-dimensional communities of bacteria surrounded by an extracellular matrix, which consists mainly of polysaccharides, proteins, extracellular DNA (eDNA), and even RNA [8,9]. The process of biofilm formation is tightly controlled in response to changing environmental cues (e.g., nutrient availability, temperature and pH variation, the nature of the surface, and fluid flow), which can be divided into three general phases: attachment, multiplication/maturation, and detachment/dispersion [3]. As biofilms are dynamic and complex biological systems, *S. aureus* has evolved a plethora of global regulators (e.g., Agr, CcpA, Sae, SarA, SigB, CodY, Rot) to fine-tune the formation, structuring, and disassembly of biofilms [2,8], implicating the complexity of *S. aureus* biofilm development. Note that these regulators mentioned above also fulfill other important functions, such as virulence and metabolism, in addition to biofilm formation.

Among the global regulators involved in *S. aureus* biofilm formation mentioned above, the catabolite control protein A (CcpA), a *trans*-acting major regulator of carbon catabolite repression (CCR) conserved in low G + C Gram-positive bacteria, modulates gene expression in response to different carbon sources [10,11,12], and has been described to be pivotal for *S. aureus* biofilm development in the presence of glucose [13]. Upon glucose availability, CcpA stimulates the upregulation of *icaA* and *cidA*, which function in polysaccharide intercellular adhesin (PIA) synthesis, and encodes a holin involved in bacterial lysis and eDNA release, respectively [14,15]. In addition to protein-based regulation network, considerable advances also unveil the important role of CcpA-controled small non-coding RNAs in *S. aureus* biofilm formation. The well-known small RNA *RNAIII*, a primary effector of the accessory gene regulator (Agr) quorum sensing system, was shown to be greatly increased in a CcpA-dependent manner when grown in glucose-rich medium [16]. Recently, a multifaceted small RNA named RsaI was identified as a CcpA-repressed small non-coding RNA in the presence of high glucose concentrations [17]. When glucose is metabolized, RsaI blocks translation by directly binding to the 3′ untranslated region (3′ UTR) of *icaR* mRNA, which encodes the transcriptional repressor of PIA production [17]. Given the unarguable importance and plentiful networks of CcpA in *S. aureus* biofilm control, a comprehensive understanding of the underlying mechanism and characterization of novel CcpA-regulated factors, if present, is of great significance to prevent biofilm-associated infections caused by *S. aureus*.

In the current study, we firstly assessed the impaired biofilm formation conferred by the *ccpA* deletion in *S. aureus* clinical isolate XN108. To investigate in greater detail the potential factors involved in CcpA-controlled biofilm, transcriptomic analysis was performed and revealed the importance of *sak*-encoding staphylokinase, a plasminogen activator with promising clinical application [18,19], in the modulation of *S. aureus* biofilm. These findings were further supported by the demonstration of direct binding of CcpA with the promoter region of *sak*, which results in decreased Sak production. Overall, this study depicts a novel model in which CcpA regulates biofilm formation through direct inhibition of *sak* expression, pinpointing Sak as a promising candidate to eradicate *S. aureus* biofilms.

## 2. Results

### 2.1. Deletion of ccpA Impairs Biofilm Formation of S. aureus Strain XN108

**Figure 1:**
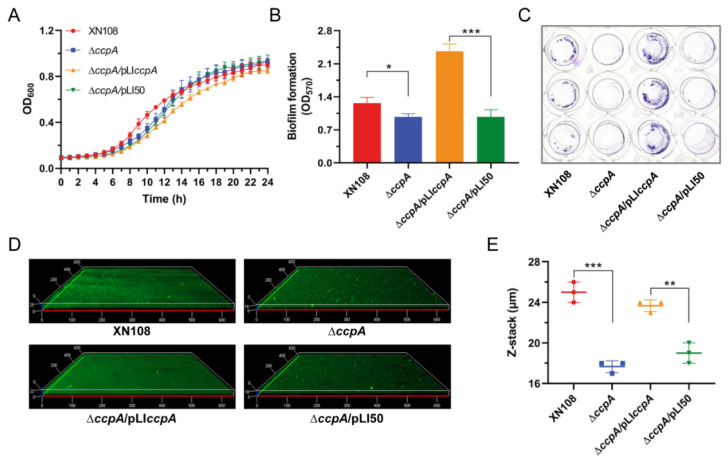
Impact of *ccpA* deletion on growth and biofilm formation of *S. aureus* strain XN108. (**A**) Profiling of bacterial growth. *S. aureus* strain XN108 and derivatives differing in *ccpA* expression were cultivated in TSB medium in triplicate at 37 °C for 24 h. Optical density at 600 nm (OD_600_) was recorded every one hour to probe bacterial growth. (**B**) Biofilm formation assessment. Biofilms were cultured in TSB + G medium under static conditions for 24 h, and quantified via crystal violet staining. The data are expressed as the mean ± SD (standard deviation) from three independent experiments. *, *p* < 0.05, ***, *p* < 0.001. (**C**) Representative images of biofilm crystal violet staining from (**B**,**D**) CLSM of biofilm formation. Biofilms were cultured in TSB + G medium under static conditions for 24 h, stained with FITC-ConA and propidium iodide (PI), and visualized by CLSM. Representative images from three independent replicates were shown. (**E**) Statistics of the biofilm thickness from (**D**) The data are expressed as the mean ± SD from three independent experiments. **, *p* < 0.01, ***, *p* < 0.001.

*S. aureus* strain XN108, a vancomycin-intermediate isolate with the vancomycin MIC of 12 µg/mL, was originally recovered from a 34-year-old steam-burned patient with wound infection [20,21]. In this study, we firstly constructed a seamless *ccpA* deletion mutant in *S. aureus* strain XN108, designated as ∆*ccpA*. When cultivated in tryptic soy broth (TSB) medium, the growth between wild-type (WT) strain XN108 and mutant ∆*ccpA* exhibited similar characteristics without a significant difference (Figure 1A). This result is in accordance with findings observed in several other *S. aureus* backgrounds, in which inactivation of *ccpA* only transiently impacted bacterial growth, but ultimately led to comparable growth yields [22,23,24]. Next, we assessed the effect of *ccpA* deletion on biofilm formation of strain XN108 by culturing the bacteria in the biofilm-promoting medium (TSB + G, TSB supplemented with 2% glucose and 2% NaCl). Crystal violet staining revealed that the biofilm formation was significantly reduced in mutant ∆*ccpA* compared with that in the WT, and the phenomenon could be restored by complementation of *ccpA* in *trans* (Figure 1B,C). Furthermore, confocal laser scanning microscopy (CLSM) demonstrated a thinner biofilm conferred by the *ccpA* deletion, with an average thickness of 25 µm and 17.7 µm for WT and ∆*ccpA*, respectively (Figure 1D,E). Upon expression of *ccpA*, the complemented strain ∆*ccpA*/pLIccpA restored biofilm with an average thickness of 23.7 µm, which is comparable to that of the WT (Figure 1D,E). Thus, inactivation of *ccpA* substantially impaired the biofilm formation of *S. aureus* strain XN108.

### 2.2. Transcriptomic Analysis Reveals Potential CcpA Regulon in S. aureus Strain XN108

**Figure 2:**
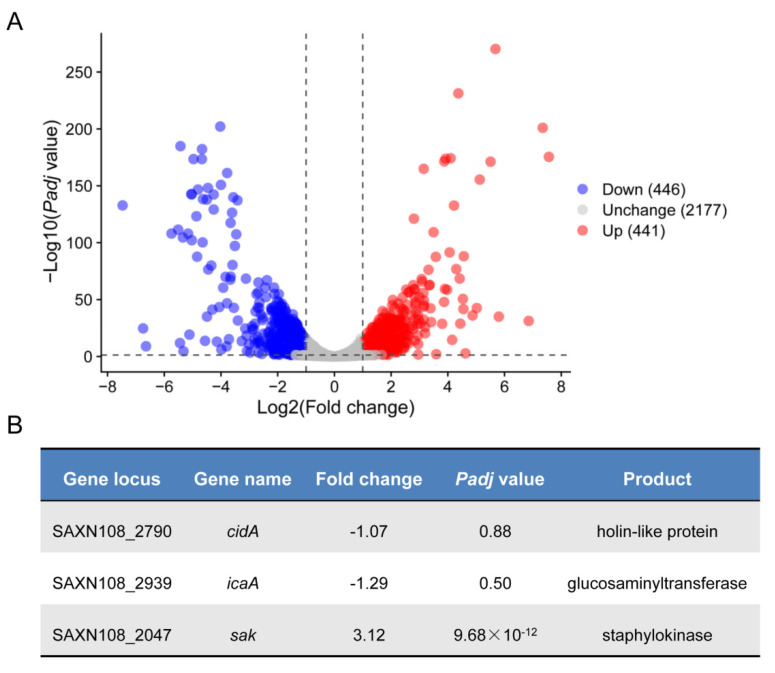
Gene expression analysis of *S. aureus* strain XN108 and mutant ∆*ccpA*. (**A**) The volcano plot of gene expression profiles revealed by RNA-seq. Downregulated differentially expressed genes (DEGs) were labeled with a blue circle, upregulated DEGs with red, and unchanged genes with gray. (**B**) Expression characteristics of selected genes.

To assess the effect of *ccpA* deletion on the gene expression of *S. aureus* strain XN108 in the genome-wide scale in detail, we extracted the total cellular RNA samples from WT and isogenic mutant ∆*ccpA*, and processed for transcriptomic sequencing (RNA-seq) in triplicate, respectively. In meet with the criteria of a |log2 (fold change)| value of >1 and a false discovery rate (FDR)-adjusted *p*-value of <0.05, a total of 887 genes were identified as differentially expressed genes (DEGs) between the WT and ∆*ccpA* mutant (Figure 2A, and Supplemental Table S1); among these, 446 genes were downregulated, and 441 were upregulated (Figure 2A, and Supplemental Table S1). The number of DEGs accounts for 29% of the entire genes encoded by XN108, demonstrating the profound role of CcpA in regulating *S. aureus* gene expression, which is in agreement with earlier findings regarding CcpA [25].

As a global regulator controlling *S. aureus* central metabolism and virulence, CcpA has been characterized to modulate *S. aureus* biofilm mainly through the *icaA* and *cidA*, which is responsible for PIA synthesis and eDNA release, respectively [14,15,26]. Interestingly, we found that transcription of both *icaA* and *cidA* did not significantly reduced in mutant ∆*ccpA* compared to WT, with only a minor fold change of 1.29 and 1.07, respectively (Figure 2B). The discrepancy combined with the impaired biofilm in mutant ∆*ccpA* led us to wonder whether CcpA could regulate biofilm formation via other factors that remain to be identified. To explore this hypothesis, we analyzed the RNA-seq data in depth, and found that *sak*, encoding the staphylokinase, was significantly increased 3.12-fold in mutant ∆*ccpA* (Figure 2B). Furthermore, it has been recognized that Sak production negatively correlates with biofilm formation, since high-Sak-producing *S. aureus* strains are often associated with less or undetectable biofilm formation in vitro [27,28]. Thus, we speculated that CcpA might control biofilm formation through a novel network involved in Sak production.

### 2.3. Deletion of ccpA Promotes the Sak Production in S. aureus Strain XN108

**Figure 3:**
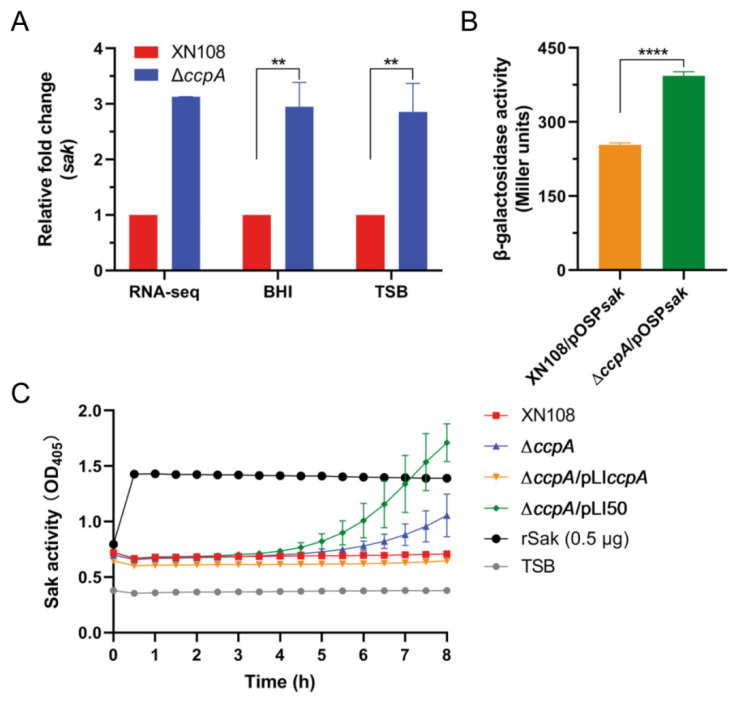
CcpA negatively regulates *sak* expression. (**A**) RT-qPCR analysis of *sak* transcription. *S. aureus* strain XN108 and ∆*ccpA* were cultivated in TSB and BHI medium, respectively. Total RNA was collected from mid-exponential phase cultures and subjected to RT-qPCR analysis. The data are expressed as the mean ± SD from three independent experiments. **, *p* < 0.01. (**B**) Promoter activity of *sak*. WT and ∆*ccpA* containing the P*sak*-*lacZ* fusion reporter plasmid were cultured in TSB medium to the mid-exponential phase, and the β-galactosidase activities were then measured. The data are expressed as the mean ± SD from three replicates. ****, *p* < 0.0001. (**C**) Measurement of Sak activity. WT and derivatives differing in *ccpA* expression were grown in TSB medium, and the activities of secreted Sak in the supernatants were detected using a chromogenic assay, in which the supernatants were incubated with human glu-plasminogen and plasmin-specific chromogenic substrate S-2251 successively, and the values for optical density at 405 nm (OD_405_) of the reactions were measured every 30 min for a total of 8 h. Plain TSB supplemented with rSak was used as positive control, and plain TSB was negative control. The data are represented as the mean ± SD from triplicate.

As mentioned above, RNA-seq indicated an increased transcription of *sak* upon *ccpA* deletion; we firstly validated this correlation using reverse transcription-quantitative PCR (RT-qPCR). Consistently, RT-qPCR results showed that the transcript level of *sak* was significantly induced in mutant ∆*ccpA* compared to WT, when cultivated to mid-exponential phase in both frequently used mediums (TSB and brain heart infusion, BHI) (Figure 3A). To further investigate the regulatory role of CcpA, we constructed the *sak* promoter-*lacZ* fusion reporter plasmid and measured the β-galactosidase activities in the WT and *ccpA* deletion mutant possessing the reporter plasmid, respectively. As shown in Figure 3B, the β-galactosidase activity of *sak* promoter was significantly increased in mutant ∆*ccpA* compared to that in the WT, with an average of 1.55-fold increase at the mid-exponential phase. Furthermore, the secretion level of protein Sak in the culture supernatants collected from WT and derivatives differing in *ccpA* expression were directly determined using a chromogenic assay. After incubation with human glu-plasminogen and plasmin-specific chromogenic substrate S-2251, the supernatants of mutant ∆*ccpA* and complemented derivative ∆*ccpA*/pLI50 containing empty-vector resulted in an obvious increase of the optical density value (Figure 3C), which correlates the activities of secreted Sak in the supernatants. In contrast, both the WT and complementary strain ∆*ccpA*/pLI*ccpA* produced undetectable Sak without optical density increase. Taken together, these results demonstrated that CcpA functions as a negative regulator of Sak production.

### 2.4. CcpA Specifically Binds to the Promoter Region of Sak

**Figure 4:**
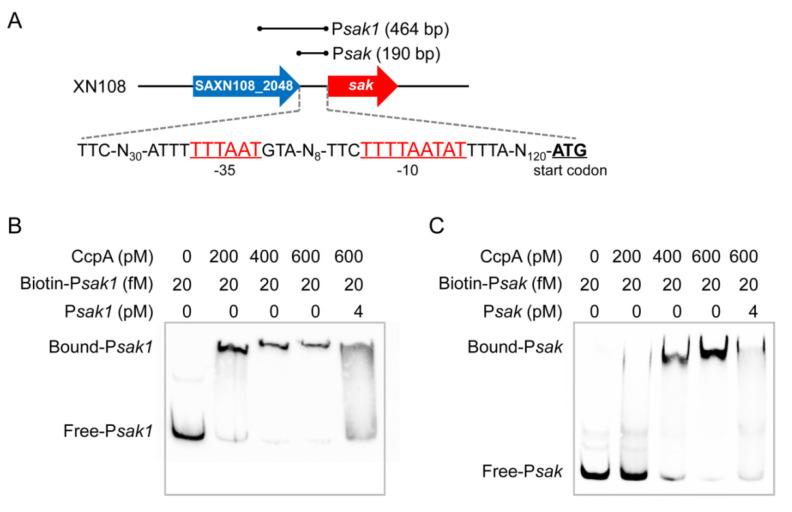
CcpA directly binds to the promoter region of *sak*. (**A**) Scheme of the gene organization and predicted promoter sequence. The −35 and −10 elements are underlined and labeled as red characters, the translational start codons are underlined and labeled as black bold characters. The location of probe P*sak* and P*sak1* is depicted above, respectively. The arrows represent transcription direction of corresponding genes that not drawn to scale. (**B**,**C**) EMSA of purified CcpA with biotin-labeled and unlabeled probe P*sak1* (**B**) and P*sak* (**C**). EMSA was conducted using increasing concentrations of CcpA (0 to 600 pM) and biotin-labeled probes (20 fM). A 200-fold excess of unlabeled fragments (4 pM) was added as cold probes.

Genomic sequence analysis of *S. aureus* strain XN108 suggested that there is a 190-bp interval between *sak* and the corresponding upstream gene. A putative promoter was further predicted in the intergenic region, which is located at 125-bp upstream of the translational start site of *sak*, with a −10 element of TTTTAATAT, and −35 element of TTTAAT (Figure 4A). Notably, CcpA is historically thought to modulate gene expression by binding *cis*-acting sequence called catabolite response elements (*cre*), which typically consists of the pseudo-palindromic motif WTGNAANCGNWNNCWW (W represents A or T, N represents one of A, G, C, T) [29]. Unfortunately, we failed to find the cognate *cre* site within both the promoter and coding regions of *sak*. To determine whether expression of *sak* is under the direct control of CcpA, we expressed and purified His-tagged recombinant protein CcpA. Then, two biotin-labeled DNA fragments, one spans the 190-bp promoter region (designated as P*sak*) and the other spans the 464-bp region upstream of *sak* (designated as P*sak1*) (Figure 4A), were amplified and used for electrophoretic mobility shift assays (EMSA), respectively. As shown in Figure 4B,C, incubation of purified CcpA protein with each of the two biotin-labeled DNA probes resulted in a dose-dependent shift in the migration of the target DNA. Furthermore, binding specificity was demonstrated by competing with a 200-fold excess of unlabeled specific competitor probes, which effectively blocked the formation of the CcpA-DNA complexes, as illustrated for both probe P*sak* and P*sak1* (Figure 4B,C). Combined, these data demonstrated that CcpA could directly control *sak* expression by specifically binding to the promoter region.

### 2.5. CcpA-Controlled Biofilm Is Involved in Direct Repression of Sak Production

**Figure 5:**
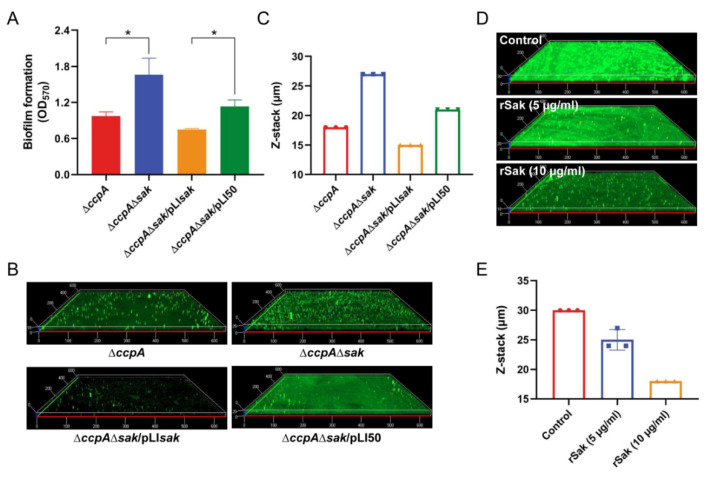
CcpA-controlled Sak regulates biofilm formation. (**A**) Assessment of biofilm formation. Biofilms were cultivated in TSB + G medium under static conditions for 24 h, and quantified via crystal violet staining. The data are expressed as the mean ± SD from three independent replicates. *, *p* < 0.05. (**B**) CLSM of biofilm formation. ∆*ccpA* and derivatives differing in Sak production were cultivated in TSB + G medium, stained with FITC-ConA and PI, and visualized by CLSM. Representative images from one of three replicates are shown. (**C**) Statistics of the biofilm thickness from (**B**) The data are expressed as the mean ± SD from triplicate. (**D**) CLSM of biofilm formation upon exogenous rSak treatment. *S. aureus* strain XN108 was cultured in TSB + G medium supplemented with different concentrations of rSak (0, 5, 10 µg/mL), and formed biofilms were assessed by CLSM. Representative images from one of three replicates are shown. (**E**) Statistics of the biofilm thickness from (**D**). The data are expressed as the mean ± SD from triplicate.

As a specific plasminogen activator, Sak has been previously shown to prevent *S. aureus* biofilm formation from attaching to surfaces, and also to facilitate the disassembly of mature biofilm in the presence of plasminogen [27]. The results of CcpA-mediated induction of Sak as demonstrated in this study, combined with the negative correlation between Sak secretion and biofilm formation, strongly suggest direct regulation of biofilm by CcpA-controlled Sak. To address this, a seamless double mutant (named ∆*ccpA*∆*sak*) lacking both *ccpA* and *sak* was firstly constructed in the mutant ∆*ccpA* background, and then *trans*-complemented (named ∆*ccpA*∆*sak*/pLI*sak*). The bacteria were cultivated in the biofilm-promoting medium TSB + G, and biofilm biomass was assessed using crystal violet staining. As shown in Figure 5A, the double mutant ∆*ccpA*∆*sak* significantly enhanced biofilm formation compared with the single mutant ∆*ccpA*. The ability of increased biofilm for ∆*ccpA*∆*sak* could be diminished by the complementation of *sak*, while not by that of empty vector (Figure 5A). Consistent with this, CLSM revealed that the double mutant ∆*ccpA*∆*sak* formed a thicker and more robust biofilm with an average thickness of 27 µm, in contrast to the thinner biofilm for ∆*ccpA* with an average thickness of 18 µm (Figure 5B,C). Upon complementation, the biofilm was significantly reduced for variant ∆*ccpA*∆*sak*/pLI*sak* but not for empty-plasmid complemented ∆*ccpA*∆*sak*/pLI50, with an average thickness of 15 µm and 21 µm, respectively (Figure 5B,C).

Previously, Sak has been recognized to interrupt biofilm formation by triggering plasminogen activation [27]. In this study, we found that biofilms varied significantly among *S. aureus* strains with distinct ability of Sak production, when cultured in TSB + G medium without addition of plasminogen (Figure 5A,B). To further demonstrate the direct linkage of Sak to biofilm formation, *S. aureus* strain XN108 was cultivated in TSB + G broth supplemented with different amounts of recombinant Sak (rSak), and the formed biofilms were analyzed by CLSM. As shown in Figure 5D, exogenous addition of rSak inhibited biofilm formation of XN108 in a dose-dependent manner. In comparison with the untreated group that formed robust biofilm with an average thickness of 30 µm, treatment with rSak at a concentration of 5 µg/mL and 10 µg/mL resulted in decreased biofilm with an average thickness of 25 µm and 18 µm, respectively (Figure 5D,E). Together, these results revealed the direct role of CcpA-controlled Sak in regulating *S. aureus* biofilm formation.

## 3. Discussion

**Figure 6:**
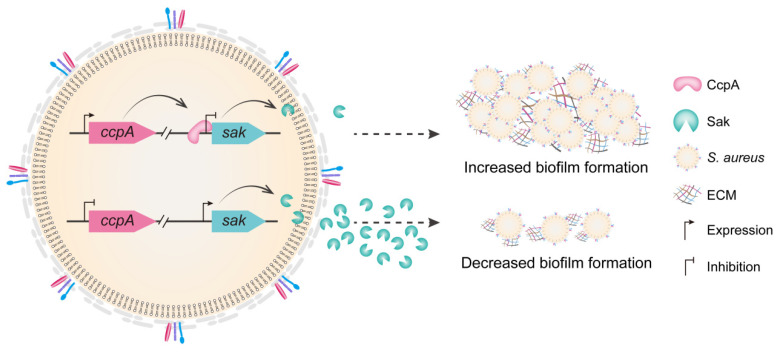
The conceptual model illustrating CcpA-controlled biofilm formation through direct repression of Sak production. CcpA directly binds to the promoter region of *sak* and inhibits *sak* transcription. In addition, overproduction of Sak impairs *S. aureus* biofilm formation either through activating plasminogen or via other cues, and the underlying mechanism remains to be fully understood. ECM, extracellular matrix.

The biofilm lifestyle plays a central role in *S. aureus* biology and pathogenesis [8]. The ability to form biofilms on both biotic and abiotic surfaces, particularly on the inert surfaces of implanted medical devices, is recognized as an important mechanism that contributes to the establishment of *S. aureus* as notorious nosocomial pathogens [7,8]. By developing biofilm, the structured extracellular matrix protects the embedded bacteria from host immune system and elicits the ineffectiveness of antibiotics, resulting in increased morbidity and mortality of human population worldwide [8,30]. Notably, the process of biofilm formation and structuring is tightly controlled, which correlated to the environmental cues and the dynamics within the bacterial community [8]. In *S. aureus*, the global regulator CcpA has been demonstrated to modulate biofilm development through induction of *cidA* and *icaA* expression, as well as by repression of the small noncoding RNA RsaI transcription [14,15,17,26]. Here, we found that CcpA directly inhibits the production of staphylokinase Sak, which ultimately affects the biofilm formation. The results of the present study revealed a novel mode of CcpA-controlled Sak in regulating *S. aureus* biofilm formation (Figure 6), and illustrated the multifaceted roles and multiple networks involving CcpA.

As the global regulator of carbon catabolite repression, CcpA belongs to the LacI repressor family and binds to a typical 14- to 16-nucleotide consensus motif called *cre* site, controlling the transcription of target genes participating in central metabolism, amino acid metabolism, and virulence [13,31]. In this study, we demonstrated that CcpA was able to bind the promoter sequence of *sak*, while we failed to identify a cognate *cre* motif. Historically, the core *cre* site was originally determined as the 16-bp consensus sequence of WTGNAANCGNWNNCWW (W represents A or T, N represents one of A, G, C, T) in *Bacillus subtilis* [29], and was recently expanded to a more flexible motif of NxTGTAAA-Yx-TTTACAMx in *Clostridium acetobutylicum*, where N and M denote bases complementary to each other, Y denotes any base, and x denotes any number [32]. The diversity and variation of identified *cre* sites indicate that the general knowledge of CcpA activity is superficial and the underlying mechanism by which CcpA executes its regulation is more sophisticated than what we know.

In *S. aureus*, several CcpA-recognized *cre* sites have been predicted and/or verified, such as the motif of TATGTAAACGTTTACACA within the promoter region of *tst* encoding the toxic shock syndrome toxin 1 (TSST-1) [24], GTATTAAACCGCTTTCATTA of *spa* encoding staphylococcal protein A (SpA) [16], ATTGTAATCGATTACATT of *hla* encoding α-hemolysin [16,33], and TAGAGAGCGTTTCCA of *cidA* encoding a holin [15], etc. Nonetheless, a majority of CcpA-regulated genes lack known *cre* sites in their promoter or protein-coding regions [23,34], in the case of *RNAIII* and *icaA*, suggesting that CcpA may indirectly regulate gene expression or recognize atypical binding sites. Corroborating these scenarios, CcpA has been shown to employ two distinct binding motifs (one is a typical *cre* site and the other is atypical) to regulate the central carbon metabolism in *Streptococcus suis* [35]. Thus, we speculate that an atypical binding site might exist in the promoter region of *sak*, which contributes to *sak* expression under the direct control of CcpA.

Gene *sak*, encoding staphylokinase that specifically converts host plasminogen to plasmin, is conserved in the majority of *S. aureus* strains [36]. However, the levels of Sak secretion vary greatly among different clinical *S. aureus* isolates, ranging from undetectable to very high amounts [27,28], and the underlying mechanism remains mysterious. In this study, Sak production was found to be significantly increased in the absence of *ccpA*, and the role of CcpA-mediated negative control of Sak production was further validated through P*sak* reporter, chromogenic assay, and EMSA. Considering that CcpA regulates gene expression in response to ever-changing environmental cues, our result provides a plausible explanation for the diverse Sak secretion phenotypes with clinical relevance.

Staphylokinase functions as a master manipulator of the host hemostatic system [37]. By triggering plasminogen activation and subsequent cleavage of host-derived fibrin, a major component of biofilm matrix in vivo, Sak has been demonstrated to prevent biofilm structuring and facilitates the detachment of mature biofilm [27]. Recently, the polymicrobial biofilms formed by *S. aureus* and *Candida albicans*, a leading fungal opportunistic pathogen, showed decreased both biofilm biomass and integrity upon Sak treatment [28]. In consistent with the phenotype, Sak significantly stimulates altered expression of several biofilm-related genes (*HWP1*, *EFG1* and *NRG1*) in *C. albicans* [28], depicting a distinct mode of Sak-controlled biofilm. In this study, we found that both over-production and exogenous addition of Sak reduce biofilm formation of *S. aureus* strain XN108, regardless of the absence of plasminogen, while the mechanism remains unknown. In fact, Sak is a cofactor for activating zymogens, and *S. aureus* could produce 12 proteases [36]. Among which, four proteases, namely the aureolysin Aur, serine protease SspA, and cysteine protease ScpA and SspB, are secreted as zymogens and require proteolytic cleavage for activation [36]. Whether Sak is able to activate the four zymogens and further modulate *S. aureus* biofilm formation and structuring remains to be discovered, and this interesting question will be the subject of our research in the future.

In summary, this study demonstrated that CcpA negatively regulates *sak* expression by direct binding to the *sak* promoter region, and revealed a novel mode for CcpA-controlled *S. aureus* biofilm. Moreover, in view of the properties of cost-effective production and lower side effects, Sak has been considered as a promising third-generation thrombolytic agent [18,19]. Combined with the anti-biofilm activity, Sak might be a potential candidate for application in the treatment for both vascular occlusion and *S. aureus*-associated biofilm infections.

## 4. Materials and Methods

### 4.1. Bacterial Strains, Plasmids, and Growth Conditions

The bacterial strains and plasmids used in this study are listed in the Supplemental Material Table S2. Unless specified otherwise, *S. aureus* strains were cultivated in tryptic soy broth (TSB; Oxoid, Basingstoke, UK) medium with shaking at 200 rpm or on tryptic soy agar (TSA) at 37 °C. When required, the antibiotic of chloramphenicol (Cm) (Sangon Biotech, Shanghai, China) was added to the *S. aureus* cultures at 10 μg/mL for plasmid selection and maintenance.

### 4.2. Construction of Gene Deletion Mutants and Complemented Strains in S. aureus

Gene allelic deletion and complementation were constructed in *S. aureus* as described previously with minor modifications [38,39,40]. To knock-out *ccpA* in *S. aureus* strain XN108, the left region and right region (~1000-bp) of *ccpA* were amplified from *S. aureus* strain XN108 genomic DNA (gDNA) using primer pairs ccpA-LAF/R and ccpA-RAF/R (see Supplemental Table S3), respectively, and ligated into the temperature-sensitive shuttle vector pBT2 via Gibson assembly master mix (NEB, Ipswich, MA, USA). The resultant vector pBT∆*ccpA* was firstly introduced into *S. aureus* strain RN4220 for modification and subsequently electroporated into strain XN108. The seamless *ccpA* deletion mutant (termed as ∆*ccpA*) was selected via homologous recombination based on the features of temperature-sensitivity and Cm resistance for plasmid pBT∆*ccpA*, and ultimately confirmed through PCR and sequencing.

For complementation of *ccpA* in mutant ∆*ccpA* background, the fragment encompassing the promoter region of *ccpA* and its coding sequence was amplified from strain XN108 gDNA with primer pair pLIccpA-F/R (Table S3), and cloned into the shuttle plasmid pLI50 to obtain pLI*ccpA* via Gibson assembly master mix (NEB, USA). The obtained vector pLI*ccpA* was successively introduced into *S. aureus* strain RN4220 and mutant ∆*ccpA*, generating the complemented derivative ∆*ccpA*/pLI*ccpA*. The empty vector pLI50 was transformed as control. The double mutant lacking *ccpA* and *sak* (termed as ∆*ccpA*∆*sak*) was constructed in the mutant ∆*ccpA* background and complemented with *sak* using a similar strategy as mentioned above.

### 4.3. S. aureus Growth Profiling

Overnight cultures of *S. aureus* strain XN108 and derivatives differing in *ccpA* expression were diluted 1:1000 into fresh TSB medium, and 200 µL of aliquots was inoculated into 96-well flat-bottomed plate (Corning, New York, NY, USA) with three replicate wells for each strain, and cultivated at 37 °C for 24 h. The values for optical density at 600 nm were measured every hour using the SmartSpecTM3000 spectrophotometer (Bio-Rad, Hercules, CA, USA).

### 4.4. Biofilm Formation

Assays were performed with minor modifications as described previously [41,42]. Briefly, overnight cultures of *S. aureus* were subcultured 1:100 into fresh TSB + G medium (TSB supplemented with 2% glucose and 2% NaCl), and used to inoculate 96-well flat-bottomed plates (Corning, Corning, NY, USA). Following 24 h of statically culturing at 37 °C, the biofilms were washed with phosphate-buffered saline (PBS, pH 7.2), stabilized with methanol, and stained with 1% crystal violet dye for 15 min. After washing with PBS (to remove planktonic cells and excess dye) and drying, 33% acetic acid was used to resolubilize the biofilms. Optical density at 570 nm (OD_570_) was quantified to represent biofilm formation.

### 4.5. Confocal Laser Scanning Microscopy (CLSM)

CLSM assays were conducted as previously described [43]. Briefly, overnight cultures of *S. aureus* diluted 1:100 in TSB + G medium were inoculated into the glass-bottom cell culture dish (15 mm in diameter; Nest, Wuxi, China), and cultivated without shaking at 37 °C for 24 h. After washing with PBS and fixed with 4% polyoxymethylene, the biofilms were stained with 50 μg/mL FITC-conjugated Concanavalin A (FITC-ConA) (Sigma-Aldrich, St. Louis, MI, USA) and 5 μg/mL propidium iodide (PI) (Sangon Biotech, Shanghai, China) at room temperature in the dark, respectively. The biofilms were then visualized with a LSM800 CLSM (Zeiss, Jena, Germany) with 488 nm excitation and 537 nm emission wavelengths for FITC-ConA, 535 nm and 615 nm for PI, respectively. A series of optical sections were observed and rendered in three-dimensional (3D) mode using ZEN 2012 lite software.

To determine the direct effect of Sak on biofilm formation, *S. aureus* strain XN108 was cultivated in TSB + G medium supplemented with rSak at a concentration of 0, 5, and 10 µg/mL, respectively. The biofilms were statically formed in the glass-bottom cell culture dish at 37 °C for 24 h, and analyzed by CLSM as mentioned above.

### 4.6. Total Cellular RNA Isolation and RNA-seq

Overnight cultures of *S. aureus* strain XN108 and mutant ∆*ccpA* were diluted 1:100 into fresh TSB and BHI medium, respectively, and cultivated with shaking at 37 °C for 6 h. The mid-exponential phase cultures were firstly lysed with lysostaphin (Sigma-Aldrich, USA) and then processed for total cellular RNA isolation using the RNAprep Pure Cell/Bacteria Kit (TIANGEN, Beijing, China) according to the manufacturer’s protocol. Following quality control, total RNA was subjected to RNA-seq library preparation and Illumina RNA sequencing conducted by a technical company (Novogene, Beijing, China). After filtering the raw sequencing reads, gene expression was determined with R package DESeq2 [44], and a gene with a |log2 (fold change)| value of >1 and a false discovery rate (FDR)-adjusted *p*-value of <0.05 was considered to be differentially expressed.

### 4.7. RT-qPCR Analysis

The total RNA isolated from mid-exponential phase cultures of *S. aureus* strain XN108 and ∆*ccpA* cultivated in both TSB and BHI medium was firstly treated with RQ1 RNase-Free DNase (Promega, Madison, WI, USA) to remove the trace gDNA contamination, and then used for cDNA synthesis using the RevertAid First Strand cDNA Synthesis Kit (Thermo Scientific, Waltham, MA, USA) in accordance with the manufacturer’s recommendation. The resultant cDNA was amplified using the TB Green™ Premix (Takara, Kusatsu, Japan) and analyzed with CFX96 Manager (Bio-Rad, Hercules, CA, USA). Three biological replicates were performed for each experimental condition. The primers used for *sak* transcript quantification are listed in Table S3. *gyrA* was used as the endogenous control for normalization, and gene expression levels were calculated by the 2^−ΔΔ*CT*^ method.

### 4.8. β-Galactosidase Activity Assay

The putative promoter was predicted in the upstream region of *sak* using the bacterial promoter recognition program BPROM (http://linux1.softberry.com/berry.phtml, accessed on 7 May 2020). To construct the *sak* promoter-*lacZ* fusion reporter plasmid, the 190-bp interval fragment encompassing the putative promoter of *sak* and the first 15-bp region of the *sak* coding sequence was amplified from *S. aureus* strain XN108 gDNA with primer pair pOSPsak-F/R (Table S3). The obtained PCR fragment was then ligated into the shuttle vector pOS1 through Gibson assembly master mix (NEB, Ipswich, MA, USA), resulting in the reporter plasmid pOSP*sak*, in which the expression of *lacZ* is under the control of *sak* promoter. The plasmid pOSP*sak* was firstly electroporated into *S. aureus* strain RN4220 for modification and ultimately introduced into XN108 and mutant ∆*ccpA*, generating the reporter strains XN108/pOSP*sak* and ∆*ccpA*/pOSP*sak*, respectively.

The β-galactosidase activity assays were conducted as previously reported [38]. Briefly, overnight cultures of the reporter strains were diluted 1:100 into fresh TSB medium containing 10 μg/mL Cm, and cultivated with shaking at 37 °C for 6 h. Bacterial cells were collected and lysed thoroughly with ABT-LSA buffer. Then, the ABT buffer and 4 mg/mL 2-Nitrophenyl-β-D-galactopyranoside (ONPG) were added to initiate the reaction. After incubation at 37 °C until a yellow color became apparent, the reactions were terminated by 1 M Na_2_CO_3_. Optical density at 420 nm (OD_420_) was determined and Miller units were calculated by the following formula: units = (1000 × OD_420_)/(T × V × OD_600_), in which T (in minutes) represents the incubation time and V (in milliliters) is the volume of bacterial culture collected.

### 4.9. Measurement of Sak Secretion

Assays were performed as described previously [45]. *S. aureus* strains differing in CcpA production were cultivated in TSB medium and the supernatants of overnight cultures were processed for Sak activity measurement. Briefly, supernatants were firstly incubated with 0.04 mg/mL human glu-plasminogen (Enzyme Research Laboratories Inc., South Bend, IN, USA) at 37 °C for 15 min, followed by addition of 3 mM plasmin-specific chromogenic substrate S-2251 (Boatman Biotech, Shanghai, China). Optical density at 405 nm of the reactions was continuously measured at 37 °C with the SmartSpecTM3000 spectrophotometer (Bio-Rad, Waltham, MA, USA). rSak (0.5 μg) was used as positive control, and plain TSB medium used as negative control.

### 4.10. Electrophoretic Mobility Shift Assay (EMSA)

The biotin-labeled DNA probes possessing the predicted promoter region of *sak* (190-bp and 464-bp) were amplified from *S. aureus* strain XN108 gDNA with 5′-biotin-labeled primers (Table S3). The obtained probes were incubated with various amounts of purified His-tagged recombinant protein CcpA in EMSA/Gel-Shift-binding buffer (Beyotime, Shanghai, China) according to the manufacturer’s instructions. After 20 min of incubation at 25 °C, the mixtures were separated in a 6% native polyacrylamide gel at 100 V, and transferred to a nylon membrane at 380 mA for 30 min in 0.5× Tris-borate-EDTA (TBE) buffer. Followed by cross-linking at 120 mJ/cm^2^ for 60 sec using a UV-light cross-linker instrument (SCIENTZ, Ningbo, China), the biotin-labeled DNA fragments were detected using the Chemiluminescent Nucleic Acid Detection Module Kit (Thermo Scientific, Waltham, MA, USA) according to the manufacturer’s suggestions, and imaged with Fusion Pulse (VILBER, Collegien, France). The unlabeled probes were added in 200-fold excess as specific competitors.

### 4.11. Statistical Analysis

Data were analyzed using GraphPad Prism v8.0 (GraphPad Software Inc., San Diego, CA, USA). For comparison of two independent data sets, Student’s *t*-tests were performed, and a *p*-value of <0.05 was considered statistically significant.

## Data Availability

The genome sequence and annotation file of *S. aureus* strain XN108 is available under GenBank accession number CP007447. The raw RNA-seq files have been deposited into the Gene Expression Omnibus (GEO) database under accession number GSE127706.

## Supplementary Materials

The following supporting information can be downloaded at: https://www.mdpi.com/article/10.3390/antibiotics11101426/s1, Table S1: Differentially expressed genes identified in mutant ∆*ccpA* compared to wild-type strain XN108; Table S2: Bacterial strains and plasmids used in this study; Table S3: Primers used in this study.
