# Supplementary material for: CcpA Regulates Staphylococcus aureus Biofilm Formation through Direct Repression of Staphylokinase Expression
Source: Antibiotics (Basel). 2022 Oct 17;11(10):1426. doi: 10.3390/antibiotics11101426 (PMC9598941; doi:10.3390/antibiotics11101426)
Supplement: Supplementary file 1 [file antibiotics-11-01426-s001.zip › Table S2. Bacterial strains and plasmids used in this study.pdf]

Table S2. Bacterial strains and plasmids used in this study.

Strains/plasmids	Characteristics[a]	Source/reference
Strains		
S. aureus strain		
RN4220	Restriction deficient cloning host	[40]
XN108	A vancomycin-intermediate strain isolated from a steam-burned patient	[20]
Δ*ccpA*	XN108 derivative with gene *ccpA* seamless deleted	This work
Δ*ccpA*/pLI*ccpA*	Δ*ccpA* complemented with gene *ccpA*, Cm^r	This work
Δ*ccpA*/pLI50	Δ*ccpA* complemented with empty vector, Cm^r	This work
Δ*ccpA*Δ*sak*	Δ*ccpA* derivative with gene *sak* seamless deleted	This work
Δ*ccpA*Δ*sak*/pLI*sak*	Δ*ccpA*Δ*sak* complemented with gene *sak*, Cm^r	This work
Δ*ccpA*Δ*sak*/pLI50	Δ*ccpA*Δ*sak* complemented with empty vector, Cm^r	This work
XN108/pOSP*sak*	XN108 derivative with plasmid pOSP*sak*, Cm^r	This work
Δ*ccpA*/pOSP*sak*	Δ*ccpA* derivative with plasmid pOSP*sak*, Cm^r	This work
E. coli strain		
DH5α	Cloning host for maintaining recombinant plasmids	Tiangen
BL21(DE3)	Expression host for exogenous protein production	Tiangen
BL21(DE3)/pET*ccpA*	Expression host for CcpA production, Kan^r	This work
Plasmids		
pBT2	Shuttle vector, temperature sensitive, Amp^r and Cm^r	[40]
pBTΔ*ccpA*	pBT2 derivative for *ccpA* deletion, Amp^r and Cm^r	This work
pBTΔ*sak*	pBT2 derivative for *sak* deletion, Amp^r and Cm^r	This work
pLI50	Expression vector, Amp^r and Cm^r	[40]
pLI*ccpA*	pLI50 derivative for *ccpA* complementation, Amp^r and Cm^r	This work
pLI*sak*	pLI50 derivative for *sak* complementation, Amp^r and Cm^r	This work
pOS1	Reporter vector with *lacZ* coding sequence for β-galactosidase assay, Cm^r	[40]
pOSP*sak*	pOS1 derivative with *lacZ* under the control of *sak* promoter, Cm^r	This work
pET28a	Expression vector, Kan^r	[39]
pET*ccpA*	Expression vector for CcpA production, Kan^r	This work

a. Cm^r, chloramphenicol resistant; Kan^r, kanamycin resistant; Amp^r, ampicillin resistant.

References

40 Rao, Y.; Peng, H.; Shang, W.; Hu, Z.; Yang, Y.; Tan, L.; Li, M.; Zhou, R.; Rao, X. A vancomycin resistance-associated WalK(S221P) mutation attenuates the virulence of vancomycin-intermediate *Staphylococcus aureus*. *J. Adv. Res*. **2022**, *40*, 167-178.

20 Zhang, X.; Hu, Q.; Yuan, W.; Shang, W.; Cheng, H.; Yuan, J.; Zhu, J.; Hu, Z.; Li, S.; Chen, W.; et al. First report of a sequence type 239 vancomycin-intermediate *Staphylococcus aureus* isolate in Mainland China. *Diagn. Microbiol. Infect. Dis*. **2013**, *77*, 64–68

39 Shang, W.; Rao, Y.; Zheng, Y.; Yang, Y.; Hu, Q.; Hu, Z.; Yuan, J.; Peng, H.; Xiong, K.; Tan, L.; Li, S.; Zhu, J.; Li, M.; Hu, X.; Mao, X.; Rao, X. β-Lactam Antibiotics Enhance the Pathogenicity of Methicillin-Resistant *Staphylococcus aureus* via SarA-Controlled Lipoprotein-Like Cluster Expression. *mBio*. **2019**, *10*, e00880-19.
