# Supplementary material for: CcpA Regulates Staphylococcus aureus Biofilm Formation through Direct Repression of Staphylokinase Expression
Source: Antibiotics (Basel). 2022 Oct 17;11(10):1426. doi: 10.3390/antibiotics11101426 (PMC9598941; doi:10.3390/antibiotics11101426)
Supplement: Supplementary file 1 [file antibiotics-11-01426-s001.zip › Table S3. Primers used in this study.pdf]

Table S3. Primers used in this study.

Primers	Sequence(5'-3')	Function
ccpA-LAF	CCGGAATTCTAGTACAACCTAGAATTGCAAC	For *ccpA* deletion
ccpA-LAR	CGGGGTACCATTCACAAAATTAGGCATT	For *ccpA* deletion
ccpA-RAF	CGCGGATCCAATTTCCTCCTTGTAAA	For *ccpA* deletion
ccpA-RAR	GCGGTCGACACGTGGTTTATCTGCTAC	For *ccpA* deletion
ccpA-LUF	ACAATATCGATTTCATGACCAACAT	For Δ*ccpA* identification
ccpA-RDR	GCGAAATCGTAAATCCAAATGAT	For Δ*ccpA* identification
ccpA-InF	CTCGGTATTCAATTCTGTGAGGTA	For Δ*ccpA* identification
ccpA-InR	ATGATGTAGCAAGAGAAGCGC	For Δ*ccpA* identification
pLIccpA-F	CGGGGTACCTTATTTTGTAGTTCCTCGGTA	For *ccpA* complementation
pLIccpA-R	CCGGTCGACTATTCCAAGGAAACTATAGACT	For *ccpA* complementation
pETccpA-F	CGCGGATCCATGACAGTTACTATATATGATGT	For *ccpA* production
pETccpA-R	CCGGTCGACTTATTTTGTAGTTCCTCGGT	For *ccpA* production
sak-LAF	AGTGCAGCGGAATTCGAGCTTACACACTTTTTTTTCAAACTG	For *sak* deletion
sak-LAR	AGGAAGCGCCAACAAAATAGTTGTTTATTATAGAAAGC	For *sak* deletion
sak-RAF	CTATTTTGTTGGCGCTTCCTCCAAATATAAT	For *sak* deletion
sak-RAR	CCTGCAGGTCGACTCTAGAGGTAGATATATTGATTTTGACGGTTG	For *sak* deletion
sak-LUF	TATGAATAGATGAAAATTTGAACAC	For Δ*sak* identification
sak-RDR	CATTTTGGGTAGCGATATTGTC	For Δ*sak* identification
sak-InF	AATGAGGTAAGTGCATCAAGTTC	For Δ*sak* identification
sak-InR	TTGTAATTAAGTTGAATCCAGGGT	For Δ*sak* identification
pLIsak-F	CGTCTTCAAGAATTCGAGCTTTCTTAGGTTAAAATGTTAAATATTT GTTAATTATTTTTTAATG	For *sak* complementation
pLIsak-R	CCTGCAGGTCGACTCTAGAGTTATTTCTTTTCTATAACAACCTTTG	For *sak* complementation
pOSPsak-F	AGCCTTAAAGACGATCCGGGTTCTTAGGTTAAAATGTTAAATAT	For P*sak-lacZ* fusion reporter
pOSPsak-R	TCACGACGTTGTAAAACGACACTTCTTTTGAGCATGGCGCTTC	For P*sak-lacZ* fusion reporter
B-Psak1-EMSAF	Biotin-TGCAAGTAAAATAACAGTTGGAAG	For biotin-labeled P*sak1* synthesis
Psak1-EMSAF	TGCAAGTAAAATAACAGTTGGAAG	For unlabeled P*sak1* synthesis
B-Psak-EMSAF	Biotin-TTCTTAGGTTAAAATGTTAAATAT	For biotin-labeled P*sak* synthesis
Psak-EMSAF	TTCTTAGGTTAAAATGTTAAATAT	For unlabeled P*sak* synthesis
Psak-EMSAR	GGCGCTTCCTCCAAATATAATA	For P*sak1*/P*sak* synthesis
sak-RTqF	TAAACCTGGGACTACACTTACA	For RT-qPCR analysis of *sak*
sak-RTqR	CGCTTGGATCTAATTCAACTACT	For RT-qPCR analysis of *sak*
gyrA-RTqF	TGGTTCAATGGATGGAGATGGC	For RT-qPCR analysis of *gyrA*
gyrA-RTqR	AGACTGACGGCTCTCTTTCA	For RT-qPCR analysis of *gyrA*
